# Contributing to achieve the goal of VISION 2020

**Published:** 2009-03

**Authors:** Peter Ackland

**Affiliations:** Chief Executive, International Agency for the Prevention of Blindness (IAPB), London School of Hygiene and Tropical Medicine, Keppel Street, London WC1E 7HT, UK.

**Figure:**
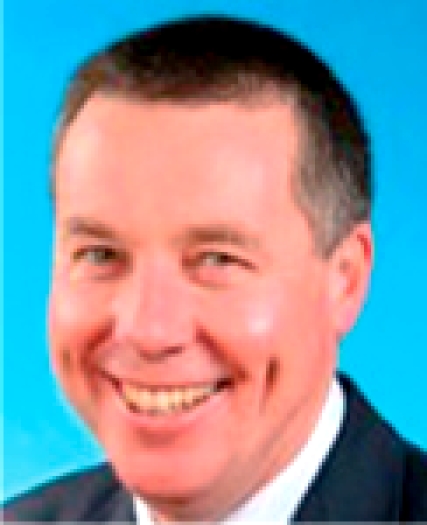


Most readers of this journal will have heard of the International Agency for the Prevention of Blindness (IAPB), but they may not be aware of exactly what the organisation does and how it functions. The purpose of this article is to give a brief overview of what IAPB does and how it contributes to the elimination of avoidable blindness in the world.

The IAPB was established in 1975 as a coordinating umbrella organisation to lead international efforts in the prevention of blindness.

IAPB presently has 97 members, which include: the major international nongovernmental development organisations (NGDOs) involved in eye health, the international professional bodies representing ophthalmologists and optometrists, universities, World Health Organization (WHO) collaborating centres, some national eye care NGDOs, and five major corporate institutions that fund VISION 2020 programmes.

IAPB's member organisations collectively deliver more than 1,500 eye health programmes, in coordination with more than 1,000 partners in over 100 countries.

IAPB is the key partner that works on the VISION 2020 initiative with WHO, in particular the WHO prevention of blindness and deafness unit (PBD). This close association, added to its knowledge of eye health programmes based upon the experience of its member organisations, means that IAPB is uniquely placed to provide strategic leadership to VISION 2020. Its work adds value and contributes to the achievement of the initiative in the following specific areas: knowledge and expertise, advocacy, promotion of VISION 2020 programmes for the prevention of blindness, and coordination.

**Figure:**
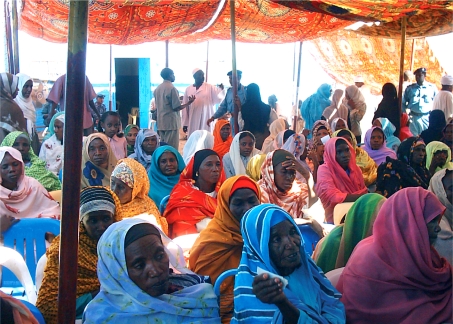
Patients waiting at an eye camp: 64% of people who are blind in the world today are women. SUDAN

Mamoun Mirghani

## Knowledge and technical expertise

IAPB provides knowledge and technical expertise to support the development of quality eye health programmes.

### VISION 2020 workshops

IAPB works in partnership with the International Centre for Eye Health (ICEH) to deliver annually more than twenty workshops that promote VISION 2020 around the world.

Topics include planning at national and district levels, specific disease control approaches, and advocacy.

So far, 150 countries have participated in these workshops and 104 have developed a national VISION 2020 plan. In the future, we hope that the IAPB regions and national coordinators for blindness control will provide us with greater input as to workshop topics and target audiences.

### Specialist committees

A number of IAPB specialist programme committees advise and promote best practice based upon the practical experience of our members' eye health programmes throughout the world.

Presently, there are committees for human resource development, technology, cataract, low vision, refractive error, and childhood blindness. Discussion groups also exist to share experience on gender, sustainability, partnership, and primary eye care.

In the future, our intention is to disseminate the work of these committees more widely, for example by creating a dedicated section on the VISION 2020 website^1^ where all key documents and information will be accessible.

## Advocacy

IAPB is involved in advocacy at different levels. It aims to achieve policy change, so that eye health can be integrated, and given greater priority, within national health care services.

### Advocating at WHO level

IAPB has worked with the IAPB East Mediterranean regional chairperson and his team and the WHO PBD unit to secure greater recognition of the importance of blindness control work within WHO structures.

Our advocacy efforts have already yielded impressive results. The 2006 World Health Assembly resolution 59.25 requested that prevention of blindness and visual impairment be added to WHO's medium-term strategic plan 2008–13, thus giving greater priority to the prevention of blindness on the global health agenda. It built on the earlier achievement of resolution 56.26 in 2003, which urged all member states to set up national plans by 2005.^2^

At the next World Health Assembly, in May 2009, it is expected that a WHO action plan for the control of avoidable blindness will be approved. This will greatly enhance the importance attached to VISION 2020 within the WHO system and will make WHO a stronger ally to promote the initiative at country level.

### Gathering evidence

IAPB also draws together the evidence that will help us to argue more effectively for prioritising resources to support VISION 2020 national plans.

For example, IAPB produced a document linking the prevention of blindness with the achievement of the United Nations' millennium development goals (MDGs),^3^ particularly the alleviation of poverty (MDG 1). This is a powerful advocacy message, which can be promoted by IAPB and its members.

### World Sight Day

IAPB promotes World Sight Day, the key date in our annual calendar to promote awareness of VISION 2020. In 2009, the main theme will be gender. In 2010, when WHO releases new figures on the prevalence of global visual impairment, the theme will be ‘State of the world's sight’. Both these themes will provide exciting opportunities for advocacy.

## Promotion of VISION 2020 programmes

IAPB has been successful in negotiating and subsequently coordinating arrangements with a number of donors to support VISION 2020 development programmes. These are then implemented by IAPB member organisations with local partners.

Such programmes include: the Standard Chartered Bank's ‘Seeing is Believing’ programme, which is providing more than US $30 million for rural and urban comprehensive eye care programmes; the Eye Fund, which will provide US $18 million to eye hospitals and IAPB members, in the form of low-interest loans, to enhance their VISION 2020 activities; the Carl-Zeiss IAPB training centre programme, which will provide US $1 million for the development of five training centres to enhance the quality of training of ophthalmic personnel; and the Optometry Giving Sight initiative, which raises funds to develop refractive error programmes.

## Coordination

The success of VISION 2020 depends on the contributions of many different stakeholders, including WHO, national and local governments, the private sector, the not-for-profit sector, and local communities. It is therefore essential to have in place:

- a good exchange of information
- coordination
- collaboration.

IAPB has a key role in ensuring that these three elements are in place and that all stakeholders work to a common agenda.

Our website,^4^ which we supplement with regular electronic newsletters to our members, is an important source of information. We also hold a Council of Members' meeting annually and a General Assembly every four years.

## Conclusion

Much has been achieved over the first ten years of VISION 2020, but a great deal more is required if we are to realise our ambition to eliminate avoidable blindness by the year 2020.

It is progress at the regional and national levels that will actually lead us to the goals of VISION 2020. Our advocacy work needs to reach a larger number of decision makers, particularly in countries and regions where the level of avoidable blindness continues to be high.

We need to attract new funding to support our aspirations to expand our regional presence and, to do this, we need to be better at communicating with potential supporters. When resources allow, we intend to ensure that the regional structure of IAPB is strengthened (see below), so that all regions have a full-time regional coordinator, as presently only the West Pacific and Latin America regions are represented.

Above all, we must continue to develop the many partnerships that already exist and craft new relationships - by working together, we can achieve so much more.

**Note from the Editors: about this issue**

**Figure:**
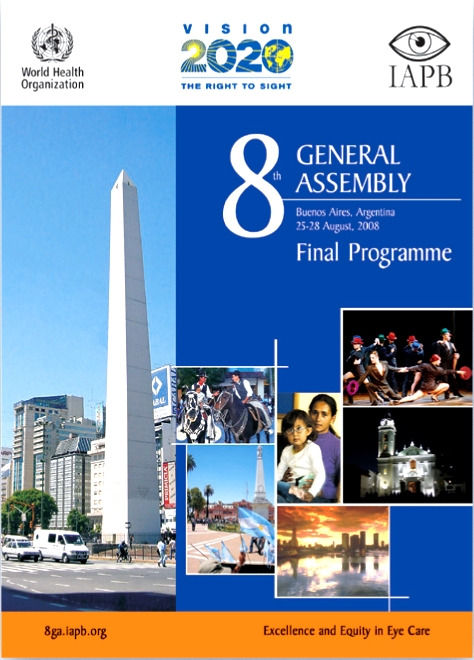
IAPB

This issue of the *Community Eye Health Journal* reports on a selection of themes discussed during the 8^th^ General Assembly of IAPB (25–28 August 2008). The articles from pages 4 to 12 do not generally contain original results from the authors; they are reports inspired by specific sessions, courses, or symposia which took place during the General Assembly. Relevant details are indicated in the blue panel at the beginning of each article.

To view the Book of Abstracts from the 8^th^ General Assembly, go to: www.v2020.org/publications-IAPB

For more information about the original presentations, email: communications@v2020.org

**Plans for strengthening the regional structure**

- Appoint a full-time coordinator in every region
- Provide better support to national. prevention of blindness committees and coordinators through training and visits.
- Make VISION 2020 programme information available through regional websites.
- Bring key stakeholders together at regional and subregional levels to share best practice and promote coordination amongst the many agencies involved.
- Encourage collaborative VISION 2020 development programmes and help to broker implementation funding for IAPB members and their partners.
- Promote a collaborative approach to our advocacy work.
